# Defining Success in Open Science

**DOI:** 10.12688/mniopenres.12780.1

**Published:** 2018-02-09

**Authors:** Sarah E. Ali-Khan, Antoine Jean, Emily MacDonald, E. Richard Gold

**Affiliations:** 1 Faculty of Law, Centre for Intellectual Property Policy (CIPP), McGill University, 3644 Rue Peel, Montreal, H3A 1W9, Canada; 2 Tanenbaum Open Science Institute (TOSI), Montreal Neurological Institute and Hospital, McGill University, 3801 University, Montreal, H3A 2B4, Canada; 3 Department of Human Genetics, McGill University, Stewart Biology Building, 1205 Avenue Dr. Penfield, Montreal, H3A 1B1, Canada

**Keywords:** Evaluation, indicators, innovation, open access, intellectual property, Open Science, policy, social and economic benefits

## Abstract

Mounting evidence indicates that worldwide, innovation systems are increasing unsustainable. Equally, concerns about inequities in the science and innovation process, and in access to its benefits, continue. Against a backdrop of growing health, economic and scientific challenges global stakeholders are urgently seeking to spur innovation and maximize the just distribution of benefits for all. Open Science collaboration (OS) – comprising a variety of approaches to increase open, public, and rapid mobilization of scientific knowledge – is seen to be one of the most promising ways forward. Yet, many decision-makers hesitate to construct policy to support the adoption and implementation of OS without access to substantive, clear and reliable evidence. In October 2017, international thought-leaders gathered at an Open Science Leadership Forum in the Washington DC offices of the Bill and Melinda Gates Foundation to share their views on what successful Open Science looks like. Delegates from developed and developing nations, national governments, science agencies and funding bodies, philanthropy, researchers, patient organizations and the biotechnology, pharma and artificial intelligence (AI) industries discussed the outcomes that would rally them to invest in OS, as well as wider issues of policy and implementation. This first of two reports, summarizes delegates' views on what they believe OS will deliver in terms of research, innovation and social impact in the life sciences. Through open and collaborative process over the next months, we will translate these success outcomes into a toolkit of quantitative and qualitative indicators to assess when, where and how open science collaborations best advance research, innovation and social benefit. Ultimately, this work aims to develop and openly share tools to allow stakeholders to evaluate and re-invent their innovation ecosystems, to maximize value for the global public and patients, and address long-standing questions about the mechanics of innovation.

## Foreword

In October 2017, thought-leaders from around the globe gathered at an Open Science Leadership Forum in the Washington DC offices of the Bill and Melinda Gates Foundation to share their views on what successful Open Science (OS) looks like. Delegates from developed and developing nations, national governments, science agencies and funding bodies, philanthropy, researchers, patient organizations and the biotechnology, pharma and artificial intelligence (AI) industries discussed the outcomes that would rally them to invest in OS, as well as wider issues of policy and implementation.

We aim to capture the breadth of this unique conversation in two reports: this first report summarizes the OS success outcomes identified at the Leadership Forum. A second longer report aimed for early Spring 2018 will address the broader topics that emerged.

Once again, we extend our sincere thanks to everyone who attended the Leadership Forum for their contributions – the scope and enthusiasm of the discussions delighted us and far exceeded our expectations.

## Context

The Leadership Forum was the first of a multi-step process to develop a ‘toolbox’ of practical and transparent indicators for assessing where and when OS models of collaboration best advance science, innovation and public benefit. This project was inspired by the recent adoption of a broad institution-wide OS policy at the Montreal Neurological Institute (the Neuro), Canada’s leading centre for neuroscience research and patient care, and one of the largest of its kind in North America. Applying this policy across all its 60-some research labs, the Neuro committed to principles of open access publication, open data and withdrew its support of patenting on any direct research outputs. Spurred by the opportunity presented by this ‘natural experiment’, McGill University created the Tanenbaum Open Science Institute (TOSI) to advance independent study of OS, and to share knowledge about its benefits, costs and mechanisms of action. Given this starting point, the focus of this project is OS in the life sciences, including disciplines and industries such as AI, that may benefit from OS collaborations in these areas. We anticipate that in coming years, our team or others may expand the indicator toolkit to assess OS practice across other scientific fields.

The next step in the process will occur on May 31 – June 1, 2018, when we will bring together experts in innovation measurement, bibliometrics, economics, sociology and other fields to translate the OS success outcomes identified at the Forum into rational and measurable indicators. Based on these conversations, we will draft an indicator toolbox over the remainder of 2018, consisting of a ‘codebook’ of indicators, their definitions, sources, qualitative methods, and associated guidance, and distribute this to stakeholders for comment. In 2019, we anticipate distributing the resulting codebook to global partners and the general public. Working with partners, we will begin to collect, analyze and openly disseminate the resulting data. Throughout this process, we invite those who attended the Forum to provide feedback on the reports and the indicators, and perhaps participate in the OS measurement and assessment activities.

This report – and the downstream development of the indicator toolbox and codebook – is funded and supported by partners with a shared interest in advancing OS: the Bill and Melinda Gates Foundation, the Wellcome Trust, the UK Government Department for Business, Energy and Industrial Strategy, and TOSI.

## Introduction

Collaborations offer the potential to not only advance basic research, but to lead to the development of new products and services on the market. In recent years, public research organizations, industry and clients, with the backing, financial support and strategic assistance of governments and philanthropy have experimented with a variety of collaborative structures.

Decreasing innovation rates and the rising costs of research and development (R&D) have increasingly led stakeholders toward open models of collaboration: ‘open science’ partnerships that rely on all or some of the pillars of open access to publications, wide sharing of data and other research outputs, and eschewing intellectual property rights (IPRs).

Theory, anecdote and early data predict that OS will accelerate discovery and innovation, maximize the value of scientific investment and bring expanding social and economic benefits. Indeed, OS has already gained significant momentum through the support of some governments, politicians and philanthropies. Yet these early supporters are working against the status quo, including entrenched business models, research culture and academic research incentives. Optimizing the outcomes of OS crucially depends on broad community adoption, which in turn depends on policy that supports and provides incentives for open practice. While some policy-makers are actively engaged in actualizing OS, the majority hesitate to enact the needed fundamental structural and cultural change in the absence of evidence. To address this legitimate concern, we are developing a toolkit upon which to build an evidence base of the benefits (and the costs) of OS, so that decision-makers in the public, private and social sectors can systematically create the conditions for success, maximize social value and spur a global transformation in how public and private partners conduct science and innovation.

In the following section, we present the hoped-for outcomes of successfully implemented OS proposed by Leadership Forum participants. These are organized by theme, each comprising a brief summary of the relevant discussion. We list the corresponding success outcomes, including scientific, clinical, social and economic factors in Appendix 1, organized by timeline – short to medium or long-term – in which we expect them to manifest.

In this document, we list the success outcomes that delegates highlighted in the Leadership Forum. Not all delegates agreed to every outcome; our goal was, instead, to capture the variety of outcomes sought rather than to reach consensus. We recognize that many, if not most, of the outcomes result from the complex interaction of factors, including OS, and therefore success or failure cannot be attributed solely to OS. However, statistical analysis and case studies will help to reveal the role that OS plays in attaining these outcomes. Nevertheless, we expect that some of them will be only aspirational and will not be measurable within a reasonable timeframe, while others are too complex or entangled with other phenomena to be measured separately. Despite this, we believe it is useful to list all outcomes, regardless of their tractability, to mark current thinking about OS and in the hopes that others will find ways to assess them in the future.

As indicated above, in the next phase of the project, we will ‘translate’ these success factors into indicators, survey scripts, case study guides and other assessment tools to identify the role of OS in contributing to each outcome. To do this, we will differentiate between success factors that act as controls (e.g., attitudes toward OS, implementation of OS, etc.), independent variables (e.g., investments in open science, access to data sharing infrastructure, etc.), and dependent variables (e.g., diversity of publications, reaching milestones along the route to introducing new products and services). While some success factors can be quantitatively measured (e.g., number of publications, number of students trained, survey results of OS attitudes), others can only be assessed qualitatively (e.g., how a partnership was created, difficulties overcome, lessons learned, etc.).

Taken together, the success outcomes listed in this document cast a wide net: at the May-June 2018 meeting, we will begin the work of translating them into indicators, and integrating these into a conceptual framework that rationalizes inputs, actors, activities, outputs, impacts and the links between them.

## Success outcomes identified at the Leadership Forum

### Increased quality and efficiency of scientific outputs

Many participants believe that OS will curb the considerable amount of waste within biomedical research and development (R&D), lowering otherwise rising costs and providing a better return on investment than presently exists. In particular, funders and philanthropy expect that OS will lead to increased reuse of data and fewer ‘throwaway’ datasets that, once used by the data generator are afterwards virtually inaccessible. Biotech and Pharma cited the ‘reproducibility crisis’, noting an urgent need for more reliable academic outputs that can be used without lengthy in-house validation. They expect that OS will build open detailed knowledge of the basic biology and biochemistry of drug targets and pathways – outcomes that point to important downstream success factors, including the ability to rapidly select the most promising drug targets, to identify failure earlier in the innovation process and to reduce costly late stage failures – all contributing to lower attrition rates within the R&D pipeline. Additionally, Biotech expects that OS will lead innovation actors to concentrate their efforts where they can excel, reducing the redundancy of roles and activities: this should further contribute to the efficiency gains that many stakeholders anticipate from OS. Most participants across sectors expect OS to generate more, and more diverse, high quality datasets, together with the meta-data necessary to use them (including descriptions of methods, reagents, protocols and workflows, the instruments or platforms used in their generation, how and why data were collected).

### Accelerated innovation and impact

Participants across sectors underlined innovation and public health-related factors as key outcomes of success. They expect OS to lead not only to faster innovation, but to deliver truly novel products and services that address unmet needs and bring measurable benefits to communities: “discovering tomorrow’s medicines, today”. Thus, government, philanthropy and national funding agencies expect improved health outcomes across their populations. Biotech and Pharma anticipate that OS will give rise to a greater diversity of research, penetrating research ‘white space’ and seeding novel research domains, including new interdisciplinary fields. Taken together, many participants expect OS to generate faster development of knowledge and its translation into products and services with marked social value.

### Increased trust in and accountability of the research enterprise

Many participants agreed that increased trust is a key success outcome, and one that is more likely to be achieved through greater openness than by other means. Many expect that OS will augment transparency, and consequently instill greater accountability across the entire research process, including at the level of reporting on the use of public research funds and the resulting public benefits. Governments expect this information to foster public trust in the research enterprise, and greater appreciation, understanding and support of science. They anticipate that this will result in increased research participation, public funding of science and private donations. In parallel with heightened transparency, many participants said that a successful implementation of OS would require new and improved mechanisms to explain research to communities and transparent governance and technical mechanisms to ensure the security of sensitive or confidential personal data while facilitating legitimate and beneficial uses.

On their side, Biotech and Pharma expect OS to augment trust between innovation actors, leading to streamlined partnering and collaboration. Again, they cited the poor reproducibility of academic outputs and a tendency for universities to compete rather than cooperate, for example by over-valuing their IP or other research outputs, which can slow partnership or knowledge transfer negotiations and fuel industry skepticism. Here again, these players believed that OS could lead to a paradigm shift toward cross-sectoral complementarity and collaboration.

### Increased equity in research

Participants highlighted increased equity as another key success factor that is most likely to be achievable by way of greater openness. They discussed equity and inclusiveness at the level of i) participation and individual agency in the research process; and ii) access to research outputs and benefits.

First, many expect that OS will foster democratization of the research enterprise, resulting in a greater diversity of people meaningfully involved and gleaning value from the process. Nevertheless, many delegates, including those from developing countries, noted that, to achieve these results, countries with the assistance of funders will first need to develop a sharing infrastructure that includes high-speed internet, as well as local research infrastructure and a critical mass of trained researchers. Bearing this in mind, many delegates believe that OS will lead to increased diversity of research leaders, collaborators and participants, including across communities of colour, gender, ethnicity and socio-economic group. Patient organizations expect OS to result in greater involvement of end-users and communities in the research process – for example, leading studies, framing research questions, making funding decisions and determining the outputs of value – and that there will be more funding available to ensure that these outputs are accessible to communities that participated in the studies. Many researchers expect OS partnerships to draw more clinical trials to OS research centres, augmenting local patient access to innovative therapeutics. Taken together, many delegates believe that OS will deliver more, and more immediate benefits, to communities from the research process.

Patient organizations also believe that greater openness will shift access and sharing decision-making to the individual donors, rather than researchers. Thus, OS will lead to new mechanisms to keep participants better informed about use of their materials and establish the individual as the ‘unit of openness’. Many participants expect OS will encourage other de-centralizations of power, including increasing collaborations in which developing country actors are equal partners or drivers of the research. In this regard, delegates underlined the need to avoid repeating historical power inequities whereby the benefits of some joint projects were coopted by the researchers from the more powerful or developed settings.

Second, by increasing access to knowledge, many participants expect OS to pave the way to increased scientific capacity in lower income, marginalized and developing communities. Again, they believe that OS will result in broader distribution of benefits, stimulating the development of research and sharing infrastructure, training, jobs and funding opportunities in lower income settings, and increasing retention of highly trained individuals in their local communities. Taken together, many participants agreed that heightened equity and inclusiveness through OS will bolster solidarity and justice, leading to greater empowerment of individuals and communities globally, and more opportunities to participate in the science innovation system, to create impact and to improve local health and well-being.

### Better opportunities and recognition of early career researchers and youth

Many participants believe that greater openness will lead to development of new high-value jobs, and better and more diverse opportunities for students, post-docs and the next generation to launch their careers. For example, some of the new roles they foresee include novel positions and pathways in academia around data management, including data scientists, curators and stewards. They also expect OS to decrease barriers to students moving between academia and industry, by increasing collaboration and knowledge flow between the two settings. Many participants strongly underlined that at the very least, OS would not disadvantage early career researchers and youth who are considering entering the sciences. However, several noted that fears about the consequences for post-docs establishing their labs are a key reason that stakeholders may hesitate to embrace OS.

### Positive economic impact

Many participants expect that OS will lead to equitable and positive economic impact. First, governments expect OS to prompt the private sector and venture capital (VC) to invest in research, where otherwise they would not. By augmenting OS public-private collaboration, OS is expected to increase the resources available to universities both through access to industry infrastructure and knowledge, and through additional funding. In particular, many participants believe that OS will lead to economic development in the communities housing OS research centres: in order for firms to take full advantage of the expertise, know-how and relationships embodied in local researchers and infrastructure, they will be obliged to set up in the environs, bringing jobs and investment. Thus, participants anticipate that OS will catalyze the development of vibrant local ecosystems, make launching start-up firms easier, and create more skilled jobs, and more jobs overall, at all levels. Industry and philanthropy further expect that OS will lead to the creation of new business models, including for VC and investment.

### Implementation success

The Leadership Forum discussions often turned toward what will be needed for a successful implementation of OS. Many times, participants across the spectrum of sectors present stated that OS will require a paradigm shift in scientific research culture in order to realize its full potential. At the same time, they said that such a transformation would, to a large degree, show that execution of OS was well underway.

As noted in previous sections, Biotech, Pharma and governments said that successful OS implementation will result in better definition of the activities and roles of the various actors within the innovation system, including their specific responsibilities in the integration of OS.

Most researchers and industry believe that OS implementation will lead to an attitudinal shift amongst researchers in favour of sharing data and collaboration: individual researchers will come to view their outputs as part of a broader initiative to build a discovery platform for the benefit for all, rather than as belonging to them. Thus, successful implementation of OS will be characterized by researchers freely sharing data, publishing by default in open access journals and avoiding the use of restrictive IPRs: open practice will be fully integrated into every-day workflow by research institutions, governments and philanthropy. To aid in this, there will be many new resources available to practitioners, including training in how to conduct open practice and manage data, and tools, such as model workflows, sharing protocols and templates.

Governments and philanthropy noted that success will be reflected in the availability of long-term and sustainable funding to support OS infrastructure and more trusted open repositories for housing research outputs.

Many participants expect there to be an increasing number of data professionals, including scientists, curators and stewards, to ensure that data are managed and put to their best use. Likewise, many said that tracking of scientific outputs by DOI or other means will become standard. In parallel, participants agreed that a critical indicator of successful OS implementation will be the recognition of a broader range of outputs as publishable material by journals, funders and institutions – including reproducibility studies, datasets, policy publications, clinical guidelines, etc. – and the assigning of value to these in promotion, tenure and funding processes.

## Next steps

 This Report serves as the basis for discussions on May 31 – June 1, 2018 in the Wellcome Trust’s London offices to start work on translating success outcomes into indicators. We anticipate that these indicators will include both quantitative and qualitative measures. We anticipate that this work will lead to the development of a toolkit consisting of indicators, a code-book of how to asses them, survey templates, and qualitative methods that we anticipate disseminating in 2019.

## Supplementary material

Supplementary file 1: Success outcomes extracted from discussions among delegates on October 5–6, 2017 in Washington, DC.

.

Supplementary file 2: Leadership forum participant list.

