# Supplementary material for: Defining Success in Open Science
Source: MNI Open Res. Author manuscript. (PMC5852639; doi:10.12688/mniopenres.12780.1)
Supplement: Supplementary file 1 [file mniopenres13840Gold-s0000.tgz › a027d2cf-a50e-41ca-a3e5-8c866e0ac066.docx]

Supplemental material 1

# We list here the success outcomes extracted from discussions among delegates on October 5-6, 2017 in Washington, DC. As noted in the Report, we opted to list a broad selection of outcomes without regard to whether they can, in practice, be measured or separately assessed. Our goal is to capture the breadth of hoped-for outcomes of OS rather than to limit discussion to only those outcomes that we know, in advance, we will be able to measure. We also note that not all delegates at the Leadership Forum agreed to all of these outcomes. Rather than being a consensus list, this represents the variety of outcomes we heard.

# Success factors

## Increased quality and efficiency of scientific outputs

### Short to medium term success outcomes:

1. Greater diversity and number of accessible, useable, and inter-operable datasets with detailed meta-data
2. Standard and consistent data management and curation of datasets
3. Greater access to negative and null results
4. Greater use, re-use and re-combination of datasets
5. Access to non-curated data (but with meta-data) to support AI approaches

### Long-term success outcomes:

1. Reduced quantity of research on the same targets or drugs
2. Increased reliability and reproducibility of scientific outputs
3. Reduced rates of scientific misconduct and retractions
4. Reduced attrition rates in the R&D pipeline
5. Reduced redundancy of roles and activities amongst players along the innovation pathway

## Accelerated innovation and impact

### Short to medium term success outcomes:

1. Faster generation and greater translation of valuable knowledge to policy and practice
2. Increased access to meaningful, understandable and useable health information
3. Increasingly diverse research questions, penetration of research ‘white space’
4. More local clinical trials

### Long-term success outcomes:

1. Faster innovation to treatments, products and services of measurable public health impact
2. Faster introduction of new clinical interventions
3. Increased diversity of clinical intervention options
4. Emergence of new inter-disciplinary fields

## Increased trust in and accountability of the research enterprise

### Short to medium term success outcomes:

1. Greater transparency across the research process
2. Improved data management practices, including marketing and stewardship
3. The establishment of effective and transparent mechanisms to govern data access
4. More effective oversight of research by government and ethics committees
5. Greater public trust, appreciation and understanding of science and the research process
6. Greater mutual trust among innovation actors

### Long-term success outcomes:

1. More and faster establishment of partnerships and collaborations with better outcomes
2. Greater diversity of partners within partnerships and collaborations
3. Greater public support of science

## Increased equity in research

### Short to medium term success outcomes:

1. Greater diversity – including across colour, gender, ethnicity, socio-economic group etc. – participating in research including as participants, collaborators and leaders
2. More international collaborations to develop infrastructure to support data-sharing and re-use
3. Increased data use outside of existing networks, including in developing countries
4. More funding and personnel to assist communities in accessing and making best use of research outputs
5. More developing country researchers, governments and NGOs involved in research and as equal partners in research collaborations
6. The development of mechanisms to enable donors to make access and sharing decisions, and keep them better informed of how their materials may be used

### Long-term success outcomes:

1. Increased development of knowledge and innovations of real value to developing country, low income and marginalized populations
2. Greater scientific capacity in developing countries, including improved research infrastructures, training, jobs and funding opportunities
3. Greater retention of highly trained individuals in their home countries or communities

## Better opportunities and recognition of early career researchers and youth

### Short to medium term success outcomes:

1. Neutral or better and more diverse opportunities for students, post-docs and young researchers to launch their careers
2. Reduced barriers to graduate students moving between academia and industry

## Positive economic impact

### Short to medium term success outcomes:

1. Increased resources available to universities through access and collaborations with industry
2. Augmented private and VC R&D investment locally
3. Increased attraction and embedding of firms in local communities
4. Greater ease in launching start-ups

### Long-term success outcomes:

1. Development of new business models to support OS and maximize public benefit
2. Creation of more skilled jobs and of new types of high value jobs at all levels
3. Increased local/regional private sector investment in R&D

## Implementation Success

### Short to medium term success outcomes:

1. More researchers engage in sharing activities such as publishing open access, freely sharing their data, and avoiding restrictive IP
2. Journals, funders and public research institutions recognize a wider range of scientific outputs as publishable material, and credit these in promotion, tenure and funding decisions
3. Greater levels of education and sharing of best practices about OS
4. More effective and comprehensive tracking of the range of scientific outputs by DOI or other means
5. Greater availability of OS resources and training for researchers, including how to conduct open practice and to manage data, model workflows, templates, protocols, etc.
6. Incorporation of OS into standard researcher workflows by research institutions, governments and philanthropy
7. Increased use of open processes and tools across the entire research workflows
8. More trusted repositories
9. Increased numbers of data professionals, including scientists, curators and stewards

### Long-term success outcomes:

1. Greater clarification by actors of their roles and responsibilities in implementing OS
2. An attitudinal shift among researchers in favour of sharing all research outputs
3. Long-term and sustainable funding available for infrastructure to support OS
